# Comparison of Transperitoneal vs. Retroperitoneal Laparoscopic Donor Nephrectomy: Impact on Quality of Life and Complications

**DOI:** 10.7759/cureus.90896

**Published:** 2025-08-24

**Authors:** Mohammed Aldawsari, Ahmed Yamani, Nassar M Alqurashi, Omar M Althobaiti, Nawaf Althobaiti

**Affiliations:** 1 Urology, Alhada Armed Forces Hospital, Al Hada, SAU; 2 College of Medicine, Taif University, Taif, SAU

**Keywords:** donor nephrectomy, hand-assisted laparoscopic donor nephrectomy, kidney transplantation, laparoscopy, quality of life (qol), retroperitoneal, transperitoneal

## Abstract

Background

Since laparoscopic donor nephrectomy (LDN) has fewer complications and a shorter recovery period than open surgery, it has become the accepted method for living kidney donation. However, the transperitoneal (TP) and retroperitoneal (RP) hand-assisted procedures are frequently employed among LDN techniques.

Objectives

At our institution, two distinct techniques for hand-assisted laparoscopic live donor nephrectomy (HALDN) are currently in use: the TP and the RP approaches. This study aims to compare these two surgical approaches across multiple clinically relevant outcomes. The primary endpoints include perioperative complication rates, postoperative pain levels, time to return to normal daily activities, postoperative renal function in donors, and donor-reported perceptions of body image following surgery.

Methods

A retrospective study of 30 donors who underwent HALDN at Alhada Armed Forces Hospital, Al Hada, Saudi Arabia, from 2024 to 2025 (11 TP and 19 RP), was conducted. Complications, creatinine levels, pain assessment, body image assessment, and quality of life (QoL) were analyzed using IBM SPSS Statistics for Windows, Version 26 (Released 2019; IBM Corp., Armonk, NY, USA).

Results

The perioperative creatinine levels in both groups were similar. The TP group only experienced an initial significant drop in serum creatinine (p = 0.024), while the RP group experienced much larger postoperative reductions in this measure (p < 0.001). Abdominal pain persisted more after surgery for RP donors (p = 0.010). However, there were no discernible variations in complications, QoL, or body image satisfaction.

Conclusion

Both TP and RP LDN methods have comparable results in terms of overall kidney function, patient satisfaction, and QoL. Although the RP method greatly improves early creatinine results, it is linked to more lasting postoperative pain. As a result, the surgical approach should be chosen individually, taking into account both patient-specific and anatomical factors.

## Introduction

Laparoscopic donor nephrectomy (LDN) is the method of choice for living kidney donation, since it is less intrusive, requires less time in the hospital, and allows for quicker recovery than open surgery [1]. Lloyd Ratner and Louis Kavoussi performed the first LDN in 1995, and the first hand-assisted laparoscopic donor nephrectomy (HALDN) was carried out in 1998 [1]. The transperitoneal (TP-LDN) and retroperitoneal (RP-LDN) methods are the two main approaches. Each approach has potential challenges as well as benefits. Furthermore, both methods have also been used with a hand-assisted technique. Hand-assistance bridges the gap between open and laparoscopic surgery by offering the potential for manual dissection, in addition to the theoretical benefit of direct tactile feedback [1]. No evidence supports one approach over another [2]. RP techniques were linked to noticeably fewer complications when the complications of the two laparoscopic techniques were compared [1]. The two approaches of HALDN - TP and RP - are currently being performed at our institution. Therefore, we aim to compare both techniques with respect to the following outcomes: perioperative complications, postoperative pain, time to return to daily activities, postoperative renal function, and donor-reported body image perception following surgery.

## Materials and methods

This study was conducted at Alhada Armed Forces Hospital, Al Hada, Saudi Arabia, and included live kidney donors who underwent HALDN between January 2024 and December 2024. The study received ethical approval from the Institutional Review Board, and all data were handled in compliance with ethical guidelines for human subject research.

A total of 30 kidney donors who underwent HALDN during the study period were included. Of these, 11 donors underwent the TP approach, and 19 donors underwent the RP approach. Two patients were excluded from questionnaire-based outcomes due to their inability to be contacted despite multiple attempts.

Inclusion and exclusion criteria

Living kidney donors were included in this study if they had been approved for donation by the institutional kidney transplant board and demonstrated normal baseline renal function, defined as a serum creatinine level within the normal reference range and an estimated glomerular filtration rate (GFR) exceeding 90 mL/min/1.73 m². Additionally, eligible donors were required to have no significant anatomical abnormalities on preoperative imaging, such as multiple renal arteries or veins, duplicated collecting systems, or other vascular or structural anomalies that could complicate the surgical procedure.

Donors were excluded from the study if they had a known history of chronic pain syndromes or psychiatric conditions that could potentially influence postoperative quality of life (QoL) assessments. Those with a prior history of major abdominal or RP surgery were also excluded due to concerns about altered anatomical planes or increased operative difficulty. Furthermore, individuals who were unable to provide informed consent, declined to participate, or could not be reached for follow-up despite multiple contact attempts were not included in the final analysis.

Data were collected retrospectively through electronic medical records and structured patient-reported outcome questionnaires. A standardized data collection form was used to retrieve the following information: demographic variables, e.g., age and gender; clinical data, including preoperative and postoperative serum creatinine; perioperative details; and surgical complications classified using the Modified Clavien-Dindo Classification System (CDCS) [3]. Postoperative pain, body image perception, and QoL were assessed using a structured questionnaire adapted from the SF-36 Health Survey [4].

All collected data were entered into Microsoft Excel (Microsoft® Corp., Redmond, WA, USA) and subsequently exported to IBM SPSS Statistics for Windows, Version 26 (Released 2019; IBM Corp., Armonk, NY, USA) for analysis. Descriptive statistics were used to summarize the data. Categorical variables were expressed as frequencies and percentages, while continuous variables were presented as means ± standard deviation or medians with interquartile ranges, depending on data distribution. Fisher’s Exact test was used to examine associations between categorical variables due to the small sample size. Independent t-tests or Mann-Whitney U tests were used to compare continuous variables between the two surgical approaches (TP vs. RP), depending on the normality of distribution. Paired t-tests were employed to assess differences in preoperative and postoperative serum creatinine levels within individuals. A p-value < 0.05 was considered statistically significant. Missing data were accounted for by reporting valid percentages.

## Results

Table 1 compares demographic and clinical variables between patients undergoing TP (n = 11) and RP (n = 19) LDN on the left side. The mean age is 38.73 years for the TP group and 34.42 years for the RP group. Gender distribution shows 33.3% males in the TP group and 66.7% in the RP group, with no statistically significant differences. Postoperative complications we looked for included, but were not limited to, fever, chest infections, atelectasis, paralytic ileus, and injury to adjacent organs. Both groups had similar creatinine levels at several time points: pre-operation (p = 0.189), post-operation (p = 0.837), two weeks (p = 0.244), and four weeks post-operation (p = 0.497).

**Table 1:** Comparison of Demographic and Clinical Variables Between Transperitoneal vs. Retroperitoneal Laparoscopic Donor Nephrectomy Groups (N = 30) CDCS, Clavien-Dindo Classification

Factor			Transperitoneal (n = 11)	Retroperitoneal (n = 19)	Total	p-value
Age (years) (N = 30)		Mean (SD)	38.73 (8.113)	34.42 (7.791)	36 (8.052)	0.162
		Min-Max	26-49	24-53	24-53	
Gender n (%)		Male	6 (33.3)	12 (66.7)	18 (60)	0.712
		Female	5 (41.7)	7 (58.3)	12 (40)	
Complication Post-operative n (%) (N = 6)	CDCS I	Chest infection	1 (33.3)	2 (66.7)	3 (50)	1
	CDCS I	Atelectasis	1 (50)	1 (50)	2 (33.3)	
	CDCS I	Paralytic ileus	1 (100)	0 (0)	1 (16.7)	
	CDCS I	Fever	0 (0)	0 (0)	0 (0)	
	CDCS II - III	Bleeding	0 (0)	0 (0)	0 (0)	
	CDCS III	Adjacent organ injury	0 (0)	0 (0)	0 (0)	
Creatinine Level Pre-operation (N = 30)		Mean (SD)	78.09 (14.488)	70.11 (16.289)	73.03 (15.886)	0.189
		Min-Max	52-103	48-98	48-103	
Creatinine Level Post-operation (N = 30)		Mean (SD)	115.55 (25.855)	113.37 (28.589)	114.17 (27.184)	0.837
		Min-Max	68-151	76-158	68-158	
Creatinine Level Two Weeks Post-operation (N = 30)		Mean (SD)	116.18 (25.992)	104.16 (27.033)	108.57 (26.857)	0.244
		Min-Max	68-154	65-142	65-154	
Creatinine Level Four Weeks Post-operation (N = 30)		Mean (SD)	110.64 (24.336)	103.63 (28.139)	106.20 (26.598)	0.497
		Min-Max	65-143	67-148	65-148	

In the RP group, the mean (SD) decrease in creatinine levels from preoperative baseline was -43.263 ± 15.165 immediately post-operation, -34.053 ± 14.447 at two weeks, and -33.526 ± 14.785 at four weeks (all p < 0.001). By comparison, the TP group showed a decrease of -37.445 ± 19.337 immediately post-operation (p = 0.024), but the reductions at two weeks (-38.091 ± 22.394, p = 0.109) and four weeks (-32.545 ± 20.447, p = 0.083) were not statistically significant, as shown in Table 2.

**Table 2:** Changes in Serum Creatinine Levels From Pre-operative Baseline at Various Post-operative Time Points in Retroperitoneal and Transperitoneal Laparoscopic Donor Nephrectomy Groups (N = 30)

Factors		Creatinine Level Post-operation	Creatinine Level Two Weeks Post-operation	Creatinine Level Four Weeks Post-operation
Retroperitoneal Laparoscopic Donor Nephrectomy Group	Mean (SD) creatinine level from pre-operative	-43.263 (15.165)	-34.053 (14.447)	-33.526 (14.785)
	p-value	<0.001	<0.001	<0.001
Transperitoneal Laparoscopic Donor Nephrectomy Group	Mean (SD) creatinine level from pre-operative	-37.445 (19.337)	-38.091 (22.394)	-32.545 (20.447)
	p-value	0.024	0.109	0.083

Post-surgery, abdominal pain rates were high in the RP group (TP: 36.3% vs. RP: 78.9%; p = 0.010). Although pain severity (<5 vs. ≥5 on a 1-10 scale) did not differ significantly (p = 0.303), the RP group experienced longer pain durations. It was the only group with persistent pain, with patients in this group also having undergone specialist referrals or further tests, as shown in Table 3.

**Table 3:** Comparison of Abdominal Pain Characteristics Before and After Surgery in Transperitoneal vs. Retroperitoneal Laparoscopic Donor Nephrectomy Groups (N = 28)

Factor		Transperitoneal	Retroperitoneal	p-value
Abdominal Pain Post-surgery	Yes	4 (21.1)	15 (78.9)	0.010
	No	7 (77.8)	2 (22.2)	
Pain Level on a Scale From 1-10	<5	3 (33.3)	6 (66.7)	0.303
	≥5	1 (10)	9 (90)	
Pain Duration Post-surgery	Less Than a Week	3 (42.9)	4 (57.1)	0.350
	More Than a Week and Less Than a Month	0 (0)	6 (100)	
	More Than a Month and Less Than Three Months	1 (20)	4 (80)	
	More Than Three Months	0 (0)	1 (100)	

Patients in both groups reported similar levels of satisfaction with their bodies after surgery. Although a higher proportion of RP patients were classified as "very satisfied" with their scars (70.6% vs. 29.4%) and tended to describe their scars more positively on the 1-10 rating scale, these differences were not statistically significant (e.g., p = 0.149 for overall scar satisfaction, and p = 0.729 for scar ratings), as shown in Table 4.

**Table 4:** Patient Satisfaction and Perceptions of Post-surgery Body Image and Scars in Transperitoneal vs. Retroperitoneal Laparoscopic Donor Nephrectomy (N = 28)

Factor		Transperitoneal	Retroperitoneal
Are you less satisfied with your body after the donation?	Never	7 (41.2)	10 (58.8)
	Little	3 (60)	2 (40)
	To a large extent	0 (0)	1 (100)
	Extreme	1 (20)	4 (80)
Do you think the procedure affects your body?	Never	10 (40)	15 (60)
	Little	1 (33.3)	2 (66.7)
	To a large extent	0 (0)	0 (0)
	Extreme	0 (0)	0 (0)
Do you feel less attractive after the donation?	Never	11 (42.3)	15 (57.7)
	Little	0 (0)	2 (100)
	To a large extent	0 (0)	0 (0)
	Extreme	0 (0)	0 (0)
Do you feel less muscular/feminine after the donation?	Never	11 (39.3)	17 (60.7)
	Little	0 (0)	0 (0)
	To a large extent	0 (0)	0 (0)
	Extreme	0 (0)	0 (0)
Do you find difficulties looking at yourself naked?	Never	11 (42.3)	15 (57.7)
	Little	0 (0)	1 (100)
	To a large extent	0 (0)	0 (0)
	Extreme	0 (0)	1 (100)
On a 1-7 scale, how satisfied are you with the surgical scar?	Very satisfied	5 (29.4)	12 (70.6)
	Mostly satisfied	5 (71.4)	2 (28.6)
	Somewhat satisfied	0 (0)	2 (100)
	Neutral, neither satisfied nor dissatisfied	1 (50)	1 (50)
	Very dissatisfied	0 (0)	0 (0)
	Mostly dissatisfied	0 (0)	0 (0)
	Somewhat dissatisfied	0 (0)	0 (0)
On a 1-7 scale, how would you describe your surgical scar?	Extremely repulsive	0 (0)	1 (100)
	A little repulsive	1 (100)	0 (0)
	Neutral, repulsive, nor beautiful	5 (50)	5 (50)
	Little beautiful	2 (66.7)	1 (33.3)
	Very beautiful	1 (16.7)	5 (83.3)
	Extremely beautiful	2 (28.6)	5 (71.4)
Rate your surgical scar on a scale from 1-10	1	0 (0)	0 (0)
	2	0 (0)	0 (0)
	3	0 (0)	1 (100)
	4	0 (0)	0 (0)
	5	1 (50)	1 (50)
	6	2 (66.7)	1 (33.3)
	7	1 (100)	0 (0)
	8	0 (0)	1 (100)
	9	1 (20)	4 (80)
	10 (completely satisfied)	6 (40)	9 (60)

Overall, there were no statistically significant differences between the TP and RP groups across various health-related QoL outcomes, including general health ratings, restrictions on strenuous and moderate activities, limitations in bending, and impacts on social activities, as shown in Table 5.

**Table 5:** Post-donation Quality of Life and Health Outcomes in Transperitoneal vs. Retroperitoneal Laparoscopic Donor Nephrectomy (N = 28)

Factor		Transperitoneal	Retroperitoneal	p-value
General health quality of life	Excellent	8 (40)	12 (60)	1
	Very good	3 (42.9)	4 (57.1)	
	Good	0 (0)	1 (100)	
	Not bad	0 (0)	0 (0)	
	Poor	0 (0)	0 (0)	
Quality of health in accordance with the year passed	Much better than it was a year ago	4 (30.8)	9 (69.2)	0.739
	Somewhat better than last year	2 (50)	2 (50)	
	Almost the same as it is	5 (50)	5 (50)	
	Somewhat worse than last year	0 (0)	1 (100)	
	Much worse than it was a year ago	0 (0)	0 (0)	
Currently, to what extent does your health condition restrict you from engaging in strenuous activities such as running, lifting heavy objects, or engaging in very strenuous sports?	Affected a lot	1 (33.3)	2 (66.7)	1
	Affected a little	7 (38.9)	11 (61.1)	
	Not affected at all	3 (42.9)	4 (57.1)	
Currently, to what extent does your health condition restrict you from engaging in moderate-intensity activities, such as moving a table, vacuuming, or gardening?	Affected a lot	2 (33.3)	4 (66.7)	0.778
	Affected a little	4 (50)	4 (50)	
	Not affected at all	5 (35.7)	9 (60.7)	
Currently, how much does your health condition limit your ability to bend, kneel, or prostrate?	Affected a lot	1 (100)	0 (0)	0.618
	Affected a little	2 (40)	3 (60)	
	Not affected at all	8 (36.4)	14 (63.6)	
During the post-donation period, to what extent did physical pain interfere with your normal activities (both indoors and outdoors)?	No conflict at all	2 (25)	6 (75)	0.220
	Very little conflict	4 (36.4)	7 (63.6)	
	Moderate conflict	5 (71.4)	2 (28.6)	
	Major conflict	0 (0)	2 (100)	
	A very major conflict	0 (0)	0 (0)	
During the period after donating, how long did you feel so depressed that nothing brought you joy?	At all times	0 (0)	0 (0)	0.522
	Most of the time	0 (0)	3 (100)	
	A lot of the time	0 (0)	1 (100)	
	Some of the time	1 (50)	1 (50)	
	A few times	2 (33.3)	4 (66.7)	
	I never felt it	8 (50)	8 (50)	
During the post-donation period, how long did you have a lot of energy?	At all times	2 (40)	3 (60)	0.783
	Most of the time	3 (33.3)	6 (66.7)	
	A lot of the time	2 (66.7)	1 (33.3)	
	Some of the time	3 (50)	3 (50)	
	A few times	1 (20)	4 (80)	
	I never felt it	0 (0)	0 (0)	
During the post-donation period, how long did you feel exhausted?	At all times	0 (0)	0 (0)	0.609
	Most of the time	1 (25)	3 (75)	
	A lot of the time	0 (0)	1 (100)	
	Some of the time	3 (75)	1 (25)	
	A few times	4 (36.4)	7 (63.6)	
	I never felt it	3 (37.5)	5 (62.5)	
During the period after donating, how long did you feel like a happy person?	At all times	7 (41.2)	10 (58.8)	0.322
	Most of the time	1 (16.7)	5 (83.3)	
	A lot of the time	2 (66.7)	1 (33.3)	
	Some of the time	1 (100)	0 (0)	
	A few times	0 (0)	1 (100)	
	I never felt it	0 (0)	0 (0)	
How true is the statement, I seem to get sick easier than others?	Undoubtedly wrong	4 (44.4)	5 (55.6)	0.439
	Probably wrong	1 (16.7)	5 (83.3)	
	I don't know	5 (62.5)	3 (37.5)	
	Probably correct	1 (25)	3 (75)	
	Undoubtedly correct	0 (0)	1 (100)	
During the post-donation period, how much time did your physical or mental health interfere with your social activities (such as visiting friends, relatives, etc)	There was conflict all the time	2 (0)	2 (100)	0.465
	There was conflict some of the time	1 (33.3)	2 (66.7)	
	There was a conflict a few times	4 (57.1)	3 (42.9)	
	There was conflict most of the time	0 (0)	3 (100)	
	There was no conflict at any time	6 (46.2)	7 (53.8)	

For the overall mean (SD) QoL and health score (out of 58), the RP group scored 45.94 (6.81), and the TP group scored 47.27 (4.54). There is no statistically significant difference between the groups (p = 0.687), indicating that both surgical approaches result in comparable overall health outcomes post-donation, as shown in Table 6.

**Table 6:** Overall Post-donation Quality of Life and Health Score Comparison in Retroperitoneal vs. Transperitoneal Laparoscopic Donor Nephrectomy (N = 28)

Factor	Mean (SD)	Median (IQR)	Min-Max	p-value
Retroperitoneal Laparoscopic Donor Nephrectomy Group	45.94 (6.814)	48 (10)	28-56	0.687
Transperitoneal Laparoscopic Donor Nephrectomy Group	47.27 (4.541)	47 (9)	41-54	

## Discussion

Living-donor nephrectomy is a distinctive surgical operation where healthy individuals voluntarily undergo surgery, accepting certain risks to help someone else - often a relative or friend. Before the advent of laparoscopic techniques, this procedure was carried out through open surgery using a flank incision and accessing the kidney via a RP method. The incidence of significant complications during this time was approximately 2% [5,6]. Recently, several laparoscopic methods have been developed for performing living-donor nephrectomies, including both TP and RP approaches, which may or may not involve hand-assisted techniques. A review indicates that the TP method is far more prevalent than the RP one, with a ratio of 9:1. The preference for this technique is attributed to advantages such as improved working space, easier navigation of abdominal anatomy via laparoscopic visualization, and potentially the influence of general surgeons, who typically favor the intraperitoneal technique [2].

Conversely, in open donor nephrectomy, the RP technique, involving a flank incision, is often preferred, as it allows for direct kidney access without breaching the intraperitoneal cavity or disturbing abdominal organs [7,8].

Our study compared both the TP and RP LDN groups, which demonstrated similar baseline demographics and preoperative renal function. Postoperatively, while overall creatinine levels were comparable at various time points, the RP group showed statistically significant reductions in creatinine immediately following surgery and at subsequent follow-ups. In contrast, the TP group exhibited a significant decrease only immediately post-operation. Additionally, the RP approach was associated with a higher prevalence and prolonged duration of postoperative abdominal pain. A review aligns with our results, indicating that the retroperitoneoscopic approach may be superior to the laparoscopic intraperitoneal approach, with fewer complications and a lower incidence of delayed graft function in patients [7]. Furthermore, another review reported significantly fewer complications in the RP group, compared to the TP group [5].

However, measures of patient satisfaction regarding pain, scars, and overall QoL did not differ significantly between groups. Our results highlight distinct short-term renal and symptomatic advantages that may inform surgical decision-making. The introduction of minimally invasive laparoscopic techniques in 1994 demonstrated that the field has evolved, and laparoscopic living-donor nephrectomy has emerged as a preferred option. This shift was driven by the numerous advantages it offers: reduced pain, improved cosmetic outcomes, quicker recovery times, and fewer complications [9,10]. The successful implementation of LDN not only brings relief to living kidney donors but also ensures that they achieve equally successful outcomes regarding kidney graft function. As research continues to illuminate the nuances of these surgical approaches, it becomes increasingly clear that the choice of technique might affect both renal recovery and overall patient experience [7,11-13].

The study has limitations, such as a small sample size, potential selection bias, and a short follow-up period that may not capture long-term outcomes. Subjective measures of pain and satisfaction also limit the generalizability of the findings. Future research should include larger, multicenter trials with longer follow-up to verify these findings and evaluate postoperative pain and renal function, using standardized assessments to clarify the benefits and drawbacks of each surgical approach.

## Conclusions

The comparison of TP and RP LDN shows that both techniques achieve similar overall renal function, QoL, and patient satisfaction. However, each method has its specific advantages. The RP approach significantly enhances early creatinine outcomes; however, it is associated with longer-lasting postoperative pain. Therefore, the choice of surgical approach should be individualized, taking into account both anatomical considerations and patient-specific factors.

## References

[1] Özdemir-van Brunschot DM, Koning GG, van Laarhoven KC, Ergün M, van Horne SB, Rovers MM, Warlé MC (2015). A comparison of technique modifications in laparoscopic donor nephrectomy: a systematic review and meta-analysis. PLoS One.

[2] He B, Bremner A, Han Y, Hamdorf JM (2016). Determining the superior technique for living-donor nephrectomy: the laparoscopic intraperitoneal versus the retroperitoneoscopic approach. Exp Clin Transplant.

[3] Dindo D, Demartines N, Clavien PA (2004). Classification of surgical complications: a new proposal with evaluation in a cohort of 6336 patients and results of a survey. Ann Surg.

[4] Ware JE Jr, Sherbourne CD (1992). The MOS 36-item short-form health survey (SF-36). I. Conceptual framework and item selection. Med Care.

[5] Simforoosh N, Basiri A, Tabibi A, Shakhssalim N, Hosseini Moghaddam SM (2005). Comparison of laparoscopic and open donor nephrectomy: a randomized controlled trial. BJU Int.

[6] Blohmé I, Fehrman I, Nordén G (1992). Living donor nephrectomy. Complication rates in 490 consecutive cases. Scand J Urol Nephrol.

[7] Wilson CH, Sanni A, Rix DA, Soomro NA (2011). Laparoscopic versus open nephrectomy for live kidney donors. Cochrane Database Syst Rev.

[8] Uehling DT, Malek GH, Wear JB (1974). Complications of donor nephrectomy. J Urol.

[9] Fonouni H, Mehrabi A, Golriz M, Zeier M, Müller-Stich BP, Schemmer P, Werner J (2014). Comparison of the laparoscopic versus open live donor nephrectomy: an overview of surgical complications and outcome. Langenbecks Arch Surg.

[10] Flowers JL, Jacobs S, Cho E (1997). Comparison of open and laparoscopic live donor nephrectomy. Ann Surg.

[11] Ahearn AJ, Posselt AM, Kang SM, Roberts JP, Freise CE (2011). Experience with laparoscopic donor nephrectomy among more than 1000 cases: low complication rates, despite more challenging cases. Arch Surg.

[12] Paragi PR, Klaassen Z, Fletcher HS (2011). Vascular constraints in laparoscopic renal allograft: comparative analysis of multiple and single renal arteries in 976 laparoscopic donor nephrectomies. World J Surg.

[13] Simforoosh N, Basiri A, Shakhssalim N, Gooran S, Tabibi A, Khoshdel A, Ziaee SA (2012). Long-term graft function in a randomized clinical trial comparing laparoscopic versus open donor nephrectomy. Exp Clin Transplant.

